# Supplementary material for: Half of resources in threatened species conservation plans are allocated to research and monitoring
Source: Nat Commun. 2020 Sep 22;11:4668. doi: 10.1038/s41467-020-18486-6 (PMC7508813; doi:10.1038/s41467-020-18486-6)
Supplement: Supplementary file 1 — Supplementary information [file 41467_2020_18486_MOESM1_ESM.pdf]

Supplementary Information

Half of resources in threatened species conservation plans are allocated to research and monitoring

Rachel T. Buxton[1*], Stephanie Avery-Gomm[2], Hsein-Yung Lin[1], Paul A. Smith[1,2], Steven J. Cooke[1,3], Joseph R. Bennett[1,3]

[1]Department of Biology, Carleton University, Ottawa, ON, Canada K1S 5B6

[2]Environment and Climate Change Canada, National Wildlife Research Centre, Ottawa, ON, Canada K1S 5B6

[3]Institute of Environmental and Interdisciplinary Science, Carleton University, Ottawa, ON, Canada K1S 5B6

[*]Corresponding author email: Rachel.buxton@colostate.edu

Supplementary Methods

Cost

All estimates of cost for management tasks (i.e., research and monitoring, RM, or action) were obtained from previously published datasets that implemented a Project Prioritization Protocol [1, 2, 3, 4] (PPP). All code and data for these calculations are available at https://figshare.com/s/c7301a5ad1da56107951. PPP is part of a systematic process intended to support cost-effective resource allocation decisions [2]. For New Zealand and New South Wales, estimates of cost and duration of each management task were obtained from expert elicitation, where experts were asked to consider a minimum set of tasks that would be required to obtain a ≥95% probability of securing each species over 50 years. For each New Zealand species, the set of tasks had compulsory management components, including outcome monitoring, services and support, project management, infrastructure, and at least one optional intervention (e.g., captive breeding, translocation; [2]). Experts estimated additional costs for outcome monitoring, which we combined with the cost for the suite of management tasks. For New South Wales, standard research and monitoring tasks were developed for all species and additional monitoring was only included if required to inform adaptive management. In New Zealand's plans, costs were estimated in 2009 current dollars. In New South Wales plans, costs were estimated in 2013 current dollars.

For U.S. terrestrial and freshwater species, Gerber et al. 2018[4] extracted the cost of each management task from species' active recovery plans, which are mandatory under the Endangered Species Act (ESA; available from the Environmental Conservation Online System, ECOS, https://ecos.fws.gov/ecp/, retrieved January 10th, 2017). About half of these data came

directly from published recovery plans (drafts and final) while the rest are entered by USFWS or NOAA biologists on a yearly basis [4]. The management tasks identified in recovery plans focus on actions that result in species no longer needing protection under the ESA and maximize the number of recovered species [5]. Recovery plans recommend site-specific recovery actions, time frames for accomplishment, and criteria for judging whether recovery has been achieved. Although active recovery plans are the best available information for U.S. threatened species management tasks we note that many plans have not been updated recently, many species do not have recovery plans, and older plans tend not to account for climate change effects. Costs from recovery plans made over a range of years (1980-2016) were converted to 2016 U.S. dollars using consumer price index values associated with the first recorded fiscal year of the management task (obtained from the Bureau of Labor Statistics [4]).

We applied a discount rate of 0.01 (*r*) per year (*t*) to calculate the net present value of future costs for each management task (*i*):

$$C_i = \sum_t^{50} \frac{c_{i,t}}{(1+r)^t} \qquad \text{Eq. 1}$$

The total cost for each management task was calculated over a 50-year period. For the U.S., this included the years the management task costs were detailed in the recovery plan (usually ~5 years) plus an extrapolated cost for the remaining duration of the last task listed (truncated to 50-years).

For all jurisdictions, we considered the cost of each management task for each species independently, such that investment in any one species management task does not change the efficacy of investment in another. Thus, for the 829 species within 564 multi-species plans in the U.S. we considered management tasks repeated for each species. We did not convert costs to a

common currency or year, because we were considering proportions of the budget allocated to RM or total budgets standardized independently for each jurisdiction.

Estimating species recovery outcomes

New South Wales: To develop recovery indices for New South Wales threatened species we used Saving our Species annual report cards from 2013-2017 (https://www.environment.nsw.gov.au/topics/animals-and-plants/threatened-species/saving-our-species-report-cards). To standardize results, we excluded species without the full set of reports. Three scores (1, 0, -1) were assigned for each year based on a colored category representing the species' overall status throughout managed sites. A score of 1 was given to species with a dark or light green category, which indicates either all managed populations are on target (stable or increasing) or threat management is on target at all sites and some populations are on target, while a few populations were not monitored. A score of 0 was given to species with an orange category, indicating population trends in some managed sites were not determined or threat management at some sites is not on target and some populations were not monitored; nevertheless, populations at remaining sites are on target. A score of -1 was assigned to species with a red or dark orange category, indicating populations at some managed sites are not on target (decreasing) or major changes are required to ensure the management is meeting its target. Sums of scores across years were used as the final recovery index.

New Zealand: To develop recovery indices for New Zealand threatened species, we used consecutive assessments (2002-2005; 2005-2008; 2008-2012) extracted from the New Zealand Threat Classification System online database (https://nztcs.org.nz/home). Similar to New South Wales, three scores were assigned (1, 0, -1) for each of the three assessment periods and we excluded species with fewer than three assessments. Change in population trend between

assessments was used to represent the change in population status because we were interested in the direct response of population dynamics after implementing management actions. For example, a score of -1 was assigned to the New Zealand rock wren (*Xenicus gilviventris*) between 2008 and 2012 assessments because the population trend changed from "decreasing 10-50%" to "decreasing 50-70%". When population trend data were unavailable for a species (or subspecies) between two consecutive assessments, changes in conservation status were used to represent the changes in population status. For example, the conservation status of the Bounty shag (*Leucocarbo ranfurlyi*) improved from "Nationally Critical" (2008) to "Nationally Endangered" (2012), and therefore, a score of 1 was assigned to represent this change of population status. Nevertheless, because the classification of conservation status also includes other information besides population trend (e.g., number of mature individuals, number and size of sub-populations, or area of occupancy) and the classification methods were revised during 2007, we further used the detailed criteria and qualifiers for each conservation status to justify our assignation for details see [6, 7]. For instance, a score of 0 was assigned to the North Island saddleback (*Philesturnus rufusater*) between 2005 ("Range Restricted") and 2008 ("Recovering") based on the qualifiers and descriptions of conservation status criteria in the government report [7].

Literature Cited

1. Bennett J. R., Maloney R., Possingham H. P. Biodiversity gains from efficient use of private sponsorship for flagship species conservation. *Proc R Soc Lond [Biol]* **282**, 20142693 (2015).
2. Joseph L. N., Maloney R. F., Possingham H. P. Optimal allocation of resources among threatened species: a project prioritization protocol. *Conserv Biol* **23**, 328-338 (2009).
3. Brazill-Boast J., *et al.* A large-scale application of project prioritization to threatened species investment by a government agency. *PLOS ONE* **13**, e0201413 (2018).
4. Gerber L. R., *et al.* Endangered species recovery: a resource allocation problem. *Science* **362**, 284-286 (2018).
5. USFWS. Endangered and threatened species listing and recovery priority guidelines. *Federal Register* **48**, 43098-43105 (1983).
6. Molloy J., *et al.* Classifying species according to threat of extinction: A system for New Zealand. Threatened species occasional publication 22. Department of Conservation, Wellington. 26 p. Accessed, (2002).
7. Townsend A., de Lange P., Duffy C., Miskelly C., Molloy J., Norton D. New Zealand Threat Classification System manual. Science & Technical Publishing. Wellington. 36p. Accessed, (2008).

Supplementary Table 1. IUCN classification of types of research and monitoring needed for endangered species. Modified from

https://www.iucnredlist.org/resources/classification-schemes

	Category	Description	Example
1	**Research**		"research", "research - remote sensing"
1.1	Taxonomy	Research into taxonomy and phylogeny	"conduct taxonomic studies", "refine taxonomy", "determine taxonomy of aguiguan population"
1.2	Population size, distribution, trends	Research into changes in population size, distribution, population trends, studies of demography. Note difference with "monitoring population trends" (3.1) below. Also different from 'how populations respond to threats/actions' (1.5, 1.6).	"conduct demographic research", "determine populations size and stage-class distribution", "assess population growth rates and viability.", "demographic study of populations", "utilize at-sea surveys to refine estimates of current population size and distribution."
1.3	Life history and ecology	Research on species habitat, genetic variability, ecology, life history	"ascertain the distribution and habitat requirements of the early blue violet and nectar source plants.", "map habitat patch distributions associated with occurrence complexes.", "conduct research necessary for species management and recovery; i.e., habitat requirements, biology, and threat analysis", "conduct biosystematic research on the species", "study genetic variability", "assess spore viability and germination requirements", "describe genome"
1.4	Harvest, use, and livelihoods	Research on harvest and use of species (and effect of harvest/use)	"determine effect of timber harvest", "track skin locations to determine source of illegal harvest"

1.5	Threats	Research examining the main threats to a species, impacts of human activities on species	"determine the correlation of land use in the st. john river corridor to lousewort distribution and abundance.", "determine disease, parasitism, predation threats", "investigate impacts of non-native predators and parasites", "clarify the threat posed by herbivory, cattle grazing, encroachment of non-native plants on t. californicum and its habitat", "conduct research necessary for species management and recovery; i.e., habitat requirements, biology, and threat analysis", "conduct toxicity tests of pesticides and other contaminants", "monitor researcher activity and impacts"
1.6	Conservation actions	Research to determine how to mitigate particular threats and/or the results and effectiveness of certain management actions	"determine population response of palila to predator control efforts", "determine effects of climate change", "study potential restoration sites", "conduct experimental habitat enhancement", "evaluation effectiveness of laws"
1.7	Public perception/attitudes	**Not in IUCN classification - research into public attitudes and knowledge of the conservation issue	"conduct a survey of public uses of bighorn sheep habitat and public attitudes regarding bighorn sheep", "assess public attitudes", "examine sociological information, such as public attitudes in and around reintroduction sites"

1.8	Coordination and prioritization of research	**Not in IUCN classification - setting research priorities, publishing, research methods, conducting research based on mgmt plans, funding research, research consultation	"convene meetings of researchers, state and federal agency personnel, and other stakeholders to evaluate progress and identify additional recovery needs", "assist in providing research opportunities and funding", "convene a meeting of all researchers."
2	**Conservation planning**		
2.1	Species action/recovery plan	Develop plans (including outlining methods, management practices, and actions) to recover species	"conduct research on the biology of the species and on suitable management tools for maintaining the natural ecosystem in which it occurs", "establish a cultivation collection monitoring and management plan", "establish delisting criteria", "develop a habitat conservation strategy for the point arena and salt point metapopulations.", "determine recovery criteria", "design mitigation plans to enhance the viability of an entire metapopulation"
2.2	Area based management plan	Develop plans (including outlining methods, management practices, and actions) for conservation areas	"develop preserve designs", "develop management guidelines for public area managers"
2.3	Harvest and trade management plan	Develop plans (including outlining methods, management practices, and actions) for harvest and trade	"develop a comprehensive trade management plan for all cacti", "develop and implement best management practices for timber harvest."
3	**Monitoring**	(more long term than 'research' - e.g., annual)	

3.1	Population trends	Monitoring of population trends (versus research into trends) - likely ongoing into the future, rather than collecting baseline information for a model	"monitor size and distribution of populations", "annually monitor abundance, population size and distribution at breeding and wintering locations.", "monitor changes in distribution and abundances of the plant and its habitat using standardized monitoring procedures at anaheim bay", "monitor population trends, habitat and distribution in new york", "develop and implement a long-term population monitoring program"
3.2	Harvest level trends	Monitor harvesting of a species (or in species habitat) and how it threatens species persistence	"continue to determine the extent and nature of timber harvest threats to shrimp."
3.3	Trade trends		"monitor interstate and international trade"
3.4	Habitat trends		"monitor changes in distribution and abundances of the plant and its habitat using standardized monitoring procedures at anaheim bay"
3.5	Threats and actions	Longterm monitorting of threats and trends	
4	**Other**		
4.1	Databases	Establish and maintain databases and data repositories	"develop and maintain a central database of survey results", "develop and maintain a gis database"

Supplementary Table 2. Parameter estimates (PE) ± standard error (SE) and 95% confidence intervals (CI), for each covariate in Beta regression models estimating the proportion of endangered species budgets allocated to research and monitoring in New South Wales (NSW), the United States (US), and New Zealand (NZ). Asterisks indicate covariates where CIs do not overlap 0, which we consider as significant effects.

Covariate	PE ± SE	2.5% CI	97.5% CI
Total budget*	-2.51 ± 0.23	-2.97	-2.05
Country: NSW	Reference category		
Country: US*	0.82 ± 0.07	0.69	0.95
Country: NZ*	0.7 ± 0.07	0.56	0.84
Taxa: Vascular plants	Reference category		
Taxa: Amphibians*	0.67 ± 0.21	0.26	1.08
Taxa: Mammals*	0.55 ± 0.13	0.29	0.80
Taxa: Bryophytes*	0.49 ± 0.24	0.02	0.95
Taxa: Birds*	-0.46 ± 0.08	-0.62	-0.29
Taxa: Fishes*	0.41 ± 0.1	0.22	0.61
Taxa: Invertebrates*	0.24 ± 0.06	0.12	0.35
Taxa: Reptiles	-0.11 ± 0.12	-0.35	0.13
Taxa: Fungus	-0.06 ± 0.17	-0.40	0.28
Benefit*	-0.14 ± 0.02	-0.19	-0.10

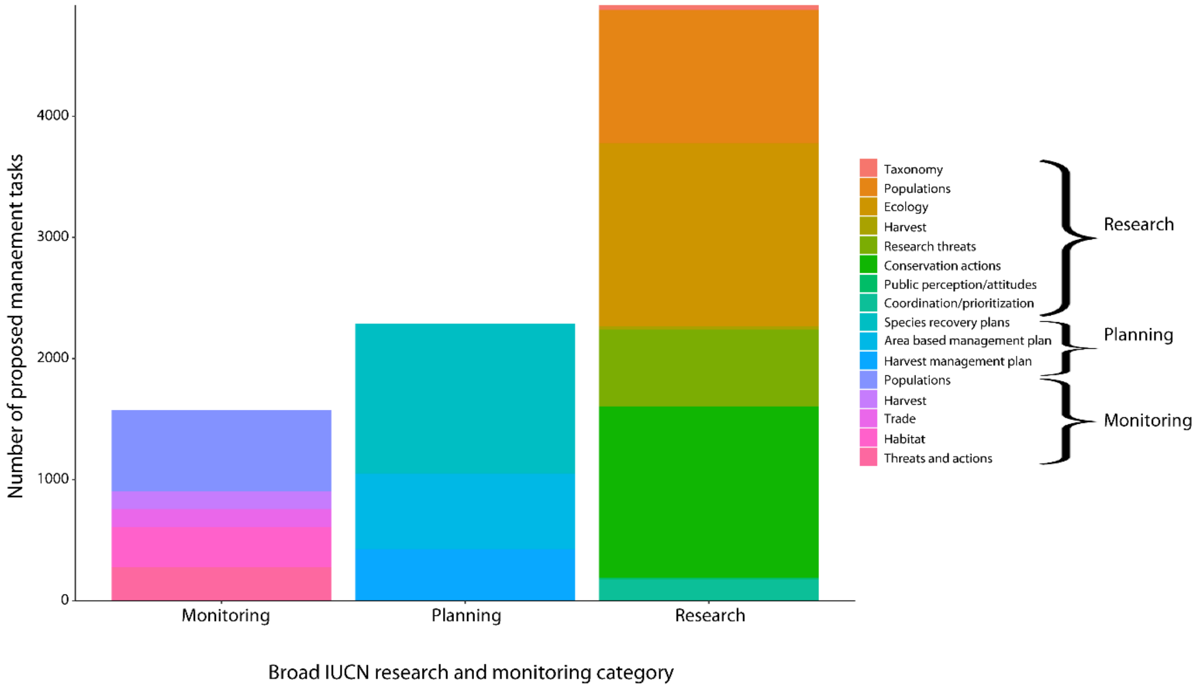

Supplementary Figure 1. For a subset of 207 US endangered species (16%), management tasks were categorized according to the IUCN criteria of monitoring, planning, and research programs.

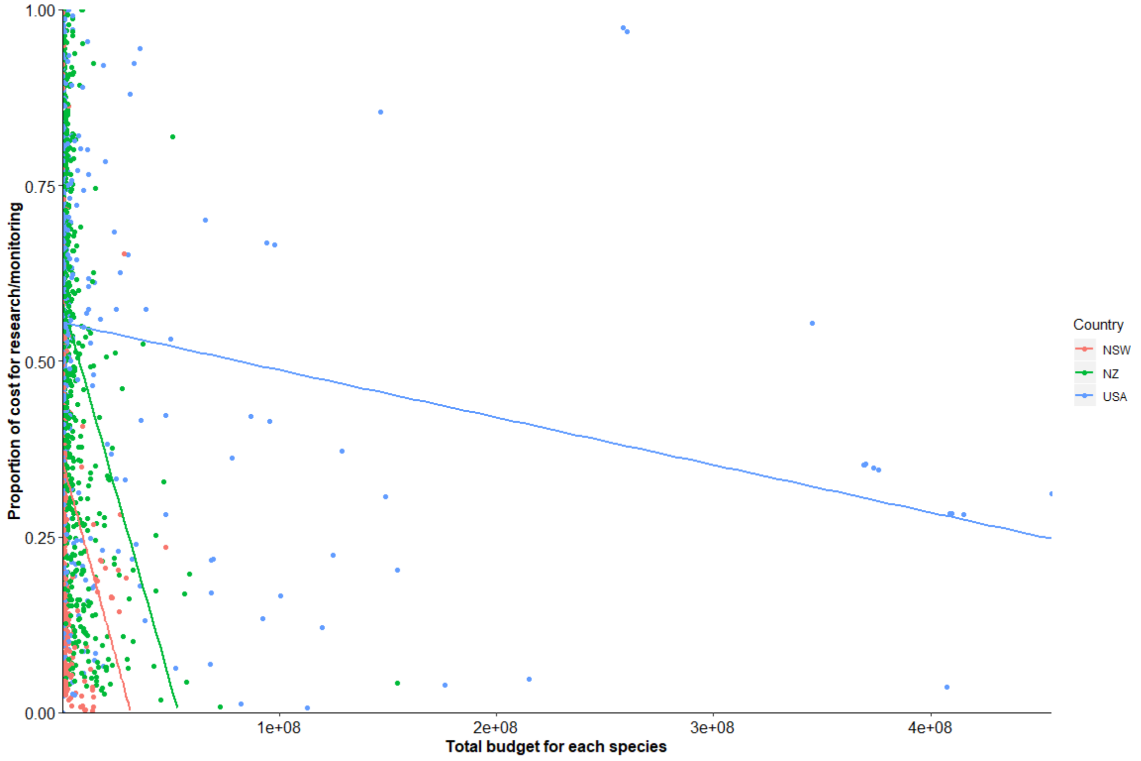

Supplementary Figure 2. A lower proportion of the budget is allocated to research and monitoring for threatened species with a higher overall proposed budget in the United States (USA), New Zealand (NZ), and New South Wales (NSW).

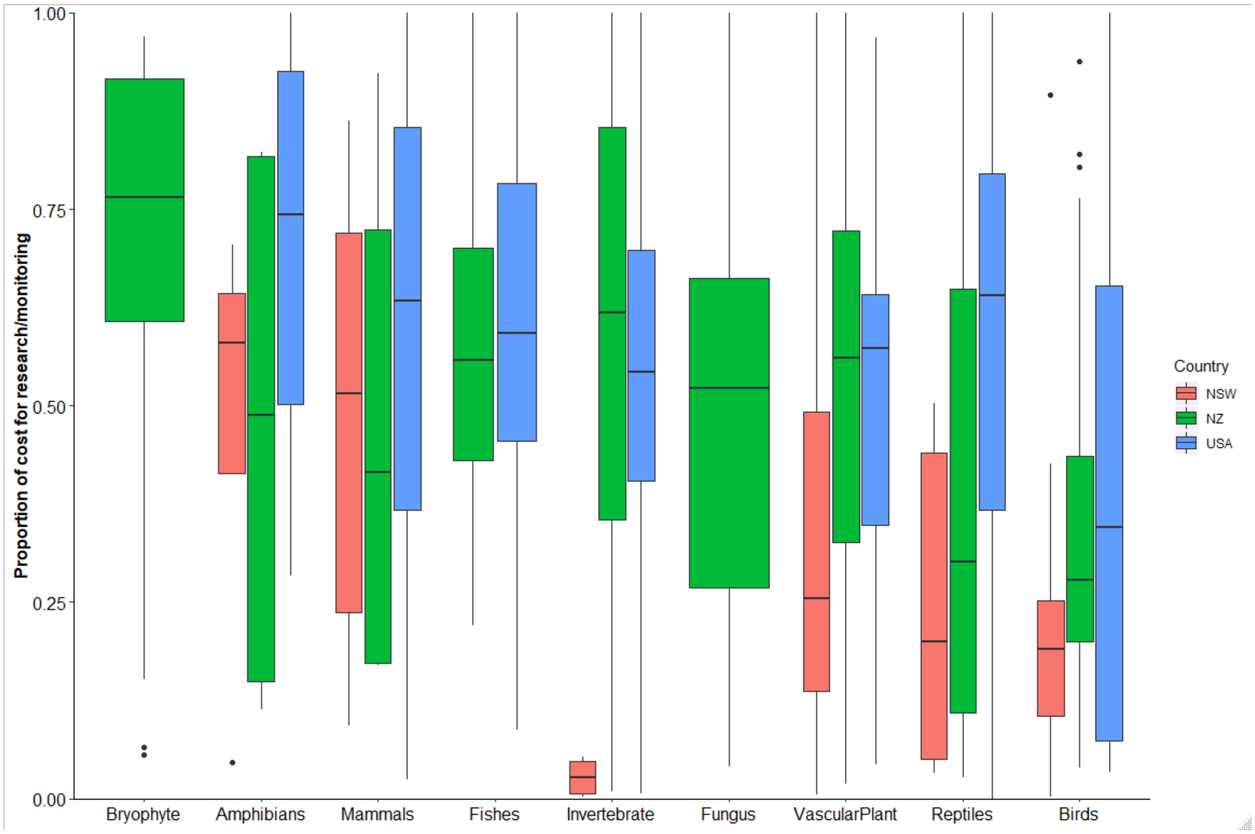

Supplementary Figure 3. The median and range of proportion of recovery plan budgets allocated to research and monitoring for n = 2255 threatened species in a variety of taxa in the United States (USA), New Zealand (NZ), and New South Wales (NSW). For each taxon, the box and whiskers show the median as a line, first and third quartiles as hinges, and the highest and lowest values within 1.5 times the interquartile range as whiskers.

Supplementary Table 3. Parameter estimates (PE) ± standard error (SE) and 95% confidence intervals (CI), for each covariate in Beta regression models estimating the proportion of endangered species budgets allocated to research and monitoring in the United States. Asterisks indicate covariates where CIs do not overlap 0, which we consider as significant effects.

**Covariate**	**PE ± SE**	**2.5% CI**	**97.5% CI**
Taxa: Flowering plants	Reference category		
Taxa: Invertebrates	-0.05 ± 0.1	-0.25	0.15
Taxa: Birds*	-0.28 ± 0.13	-0.53	-0.03
Taxa: Fishes	0.12 ± 0.13	-0.13	0.37
Taxa: Nonflowering plants	0.21 ± 0.2	-0.19	0.61
Taxa: Amphibians*	0.61 ± 0.28	0.07	1.15
Taxa: Reptiles	0.08 ± 0.23	-0.38	0.54
Taxa: Mammals	0.2 ± 0.15	-0.1	0.5
Status: Not listed	-0.41 ± 0.4	-1.19	0.38
Status: Threatened	0.04 ± 0.09	-0.14	0.22
Status: Endangered	Reference category		
Proportion of RM tasks completed	-0.03 ± 0.04	-0.1	0.04
Recovery potential	-0.03 ± 0.04	-0.1	0.04
Benefit	0 ± 0.03	-0.07	0.06
Proportion of RM tasks assigned high priority*	0.19 ± 0.03	0.12	0.26
First fiscal year of earliest RM*	-0.05 ± 0	-0.06	-0.05
Number of species in a multi-species plan	0.04 ± 0.04	-0.03	0.11

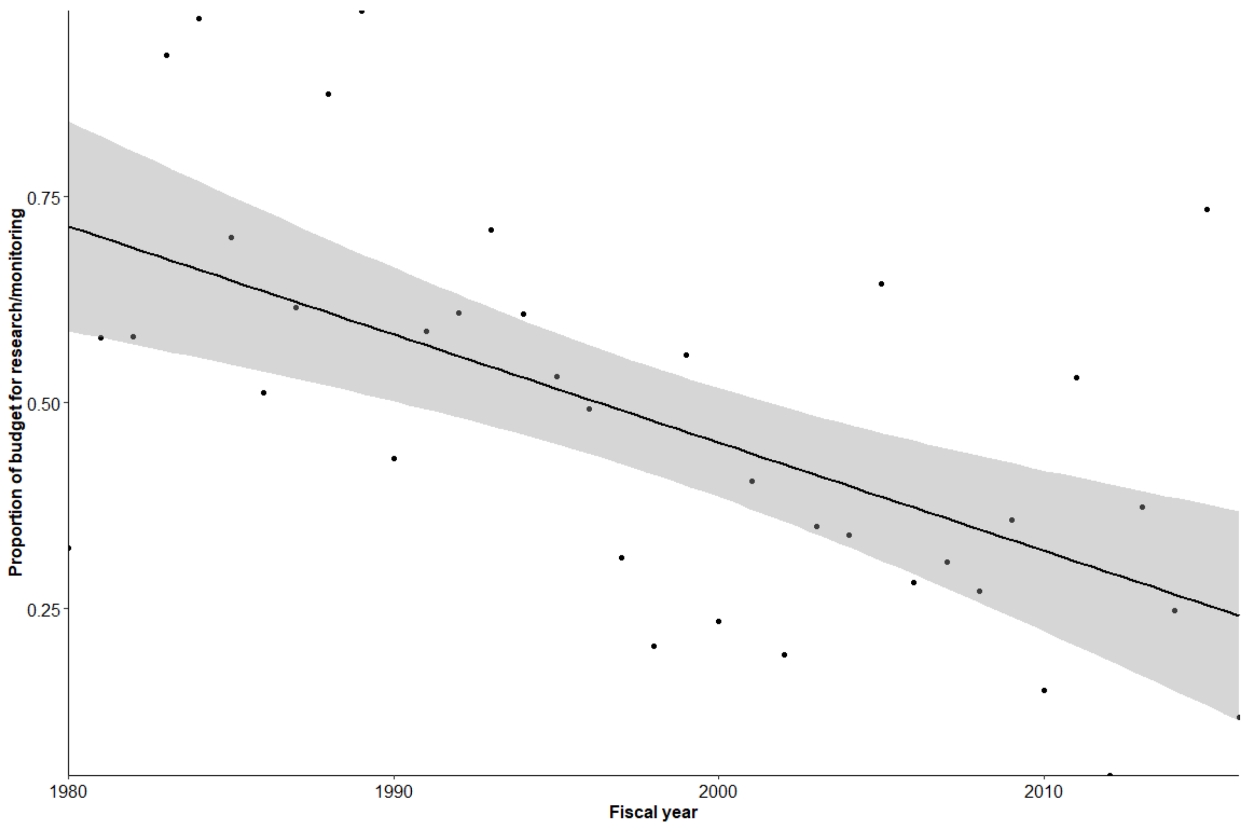

Supplementary Figure 4. A higher proportion of the budget is allocated to research and monitoring for threatened species with a recovery plan that began earlier in the United States. The shaded error band represents the standard error around a linear model fit between x and y variables.
