# Supplementary material for: Half of resources in threatened species conservation plans are allocated to research and monitoring
Source: Nat Commun. 2020 Sep 22;11:4668. doi: 10.1038/s41467-020-18486-6 (PMC7508813; doi:10.1038/s41467-020-18486-6)
Supplement: Supplementary file 3 — Reporting Summary [file 41467_2020_18486_MOESM3_ESM.pdf]

nature research

Corresponding author(s): Rachel T. Buxton

Last updated by author(s): Aug 14, 2020

# Reporting Summary

Nature Research wishes to improve the reproducibility of the work that we publish. This form provides structure for consistency and transparency in reporting. For further information on Nature Research policies, see Authors & Referees and the Editorial Policy Checklist.

## Statistics

For all statistical analyses, confirm that the following items are present in the figure legend, table legend, main text, or Methods section.

| n/a | Confirmed | |
|---|---|---|
| ☐ | ☒ | The exact sample size (*n*) for each experimental group/condition, given as a discrete number and unit of measurement |
| ☐ | ☒ | A statement on whether measurements were taken from distinct samples or whether the same sample was measured repeatedly |
| ☐ | ☒ | The statistical test(s) used AND whether they are one- or two-sided<br>*Only common tests should be described solely by name; describe more complex techniques in the Methods section.* |
| ☐ | ☒ | A description of all covariates tested |
| ☐ | ☒ | A description of any assumptions or corrections, such as tests of normality and adjustment for multiple comparisons |
| ☐ | ☒ | A full description of the statistical parameters including central tendency (e.g. means) or other basic estimates (e.g. regression coefficient) AND variation (e.g. standard deviation) or associated estimates of uncertainty (e.g. confidence intervals) |
| ☐ | ☒ | For null hypothesis testing, the test statistic (e.g. *F*, *t*, *r*) with confidence intervals, effect sizes, degrees of freedom and *P* value noted<br>*Give P values as exact values whenever suitable.* |
| ☒ | ☐ | For Bayesian analysis, information on the choice of priors and Markov chain Monte Carlo settings |
| ☒ | ☐ | For hierarchical and complex designs, identification of the appropriate level for tests and full reporting of outcomes |
| ☒ | ☐ | Estimates of effect sizes (e.g. Cohen's *d*, Pearson's *r*), indicating how they were calculated |

*Our web collection on statistics for biologists contains articles on many of the points above.*

## Software and code

Policy information about availability of computer code

| | |
|---|---|
| Data collection | Data were collected from previously published Access databases and USFWS databases. No specialized software was required |
| Data analysis | All data analysis was performed in R version 3.6.1. All code will be made freely available at https://doi.org/10.6084/m9.figshare.12071358.v1 |

For manuscripts utilizing custom algorithms or software that are central to the research but not yet described in published literature, software must be made available to editors/reviewers. We strongly encourage code deposition in a community repository (e.g. GitHub). See the Nature Research guidelines for submitting code & software for further information.

## Data

Policy information about availability of data

All manuscripts must include a data availability statement. This statement should provide the following information, where applicable:

- Accession codes, unique identifiers, or web links for publicly available datasets
- A list of figures that have associated raw data
- A description of any restrictions on data availability

The datasets generated during and/or analysed during the current study are available in the Figshare repository, https://doi.org/10.6084/m9.figshare.12071358.v1. Note that some unique U.S. species identifiers have been removed in compliance with USFWS. Data for recovery indices in New Zealand were extracted from the NZ Threat Classification System online database (https://nztcs.org.nz/home), for New South Wales from Saving our Species (https://www.environment.nsw.gov.au/topics/animals-and-plants/threatened-species/saving-our-species-report-cards), and for the United States from https://www.pnas.org/content/pnas/suppl/2016/03/08/1525085113.DCSupplemental/pnas.1525085113.sapp.pdf.

# Field-specific reporting

Please select the one below that is the best fit for your research. If you are not sure, read the appropriate sections before making your selection.

☐ Life sciences ☐ Behavioural & social sciences ☒ Ecological, evolutionary & environmental sciences

For a reference copy of the document with all sections, see nature.com/documents/nr-reporting-summary-flat.pdf

# Ecological, evolutionary & environmental sciences study design

All studies must disclose on these points even when the disclosure is negative.

| | |
|---|---|
| Study description | We assessed the proportion of the budget allocated to research or monitoring for a total of 2328 species, independently managed subspecies, or distinct populations: 700 in New Zealand, 361 in New South Wales, and 1267 species in the U.S. We explore the effect of species characteristics on the proportion of the budget allocated to research and monitoring using a beta regression modelling approach. |
| Research sample | Our research sample was all threatened species in New Zealand, New South Wales, and the United States from previously existing data sets. For New Zealand (700 species) and New South Wales (361 species) the data were obtained from Bennett et al 2015 (10.1098/rspb.2014.2693) and for the US (1267 species), data were obtained from Gerber et al 2018 (10.1126/science.aat8434). |
| Sampling strategy | Because we used previously published data sets, no power calculation was performed. Also because we used all threatened species from each jurisdiction no power calculation was necessary. |
| Data collection | Previously published databases of cost of management tasks for threatened species were gathered in mid-2019. We extracted previously published recovery indices for the U.S. (Gerber 2016, 10.1073/pnas.1525085113), and from recurring species assessments in New Zealand (https://nztcs.org.nz/home) and New South Wales (https://www.environment.nsw.gov.au/topics/animals-and-plants/threatened-species/saving-our-species-report-cards). Two authors (RTB and HYL) extracted full sets of threatened species reports over 3 reporting periods for NZ (2002-2005; 2005-2008; 2008-2012) and 5 reporting periods for NSW (2013-2017). Three scores (1, 0, -1) were assigned to each assessment period based on: NSW) a colored category representing the species overall status throughout managed sites, and NZ) change in population trend between assessments. |
| Timing and spatial scale | We collected all these data last year (2019). The data were from previously published databases of cost for management tasks of threatened species (2009: New Zealand, 2013: New South Wales, and 2016: United States). To collect recovery outcomes, we used previously published data from reports to congress in the United States and similar data collated from semi-annual recovery reports for New Zealand and New South Wales. |
| Data exclusions | We excluded 2 extinct species from further analysis (as there are no management 'actions' that can be performed for extinct species, thus skewing the research/monitoring proportions). We also removed 6 species with budgets over 5 times the median to improve model fit. We note that results are robust to the inclusion or exclusion of these species. Exclusion criteria were established before analyses were performed. |
| Reproducibility | This was not an experimental study, we acquired data from published recovery strategies for threatened species. However, we have described all analyses at length in the methods and an extended supplementary methods to ensure that the analytic methods are reproducible. |
| Randomization | We examined the proportion of the budget allocated to research/monitoring versus action for threatened species based on costs of management tasks in recovery plans. We used each species as a unit, thus no randomization was required. |
| Blinding | Blinding was not relevant to our study. Two observers (including the first author) scored the methods description of each management task for threatened species as "action" or "research/monitoring". We used defined conservation action and research/monitoring categories from the International Union for Conservation of Nature, so there was little potential for observer bias. |

Did the study involve field work? ☐ Yes ☒ No

# Reporting for specific materials, systems and methods

We require information from authors about some types of materials, experimental systems and methods used in many studies. Here, indicate whether each material, system or method listed is relevant to your study. If you are not sure if a list item applies to your research, read the appropriate section before selecting a response.

## Materials & experimental systems

| n/a | Involved in the study |
|---|---|
| ☒ | ☐ Antibodies |
| ☒ | ☐ Eukaryotic cell lines |
| ☒ | ☐ Palaeontology |
| ☒ | ☐ Animals and other organisms |
| ☒ | ☐ Human research participants |
| ☒ | ☐ Clinical data |

## Methods

| n/a | Involved in the study |
|---|---|
| ☒ | ☐ ChIP-seq |
| ☒ | ☐ Flow cytometry |
| ☒ | ☐ MRI-based neuroimaging |
